# Decoding Epigenetic Switches: How Histone Acetylation/Deacetylation Regulates Mononuclear/Macrophage Fate in Bone Disorders

**DOI:** 10.7150/ijbs.125544

**Published:** 2026-01-01

**Authors:** Li Xu, Chen Shen, Xin Liu, Yi Qin, Zebin Wu, Ziyu Zhang, Qifeng Sheng, Qihan Wang, Feng Zhou, Gaoran Ge, Dechun Geng

**Affiliations:** Department of Orthopedics, The First Affiliated Hospital of Soochow University, Shizi Street, Suzhou, Jiangsu, China.

**Keywords:** orthopedic diseases, bone homeostasis, mononuclear phagocyte, histone acetylation

## Abstract

Monocyte-macrophage plays a central role in innate immunity, tissue homeostasis maintenance, and disease progression. These phagocytes, which originate from blood monocytes or embryonic sources, are imperative for inflammatory responses, tissue repair, and bone remodeling. In orthopedic diseases, including osteoarthritis, rheumatoid arthritis, osteoporosis, and fractures, changes in histone acetylation are key to regulating macrophage gene expression, polarization, differentiation into osteoclasts, and pathological bone remodeling. Histone acetylation (mediated by histone acetylases) and deacetylation (mediated by histone deacetylases) directly influence important transcription factors in the monocyte-macrophage system by dynamically modulating chromatin accessibility. This review systematically examines the epigenetic network involving histone acetylation and deacetylation monocyte-macrophage, exploring its translational potential in bone-related diseases.

## Introduction

As a key component of innate immunity, the monocyte-macrophage system plays a dual role in the pathophysiology of orthopedic diseases. This system not only regulates bone balance via osteoclastogenesis, a process involving the specific differentiation of bone marrow-derived monocytes (BMDM), but also maintains typical macrophage features that facilitate inflammatory responses, repair, and homeostasis. Recent research suggests that its function is closely linked to epigenetic regulation^1^, ^2^. Histone acetylation considerably affects macrophage-mediated inflammatory cytokine secretion^3^, osteoclast (OC) differentiation, and bone metabolism balance by dynamically modifying the chromatin structure. The imbalance between osteoporosis (OP) and fracture healing is often accompanied by excessive activation of OCs, which are derived from the monocyte-macrophage system and depend on histone acetylation regulated by enzymes such as ATP-citrate lyase (ACLY). In osteoarthritis (OA) and rheumatoid arthritis (RA), the proinflammatory phenotype of the synovial macrophages (M1) is directly related to increased acetylation levels of the tumor necrosis factor α (TNF-α) and interleukin 1β (IL-1β) promoter regions^4^. Abnormal histone deacetylase (HDAC)-mediated histone deacetylation may exacerbate inflammatory bone destruction in ankylosing spondylitis (AS)^5^. While targeting histone acetylation, such as with HDAC inhibitors, has demonstrated potential for modulating inflammation and aiding repair in some orthopedic disease models, the cell-specific mechanisms and safety of clinical use remain to be thoroughly examined. This article reviews the histone acetylation regulatory network within the monocyte-macrophage system, exploring its molecular mechanisms and therapeutic potential for osteoporosis (OP), osteoarthritis (OA), rheumatoid arthritis (RA), and bone repair. The goal is to offer a new perspective for precise intervention in orthopedic diseases.

## 1. Classification and function of histone acetyl transferases (HATs) and deacetylases (HDACs)

In recent years, the role of epigenetic regulation (including histone modification, DNA methylation, and non-coding RNA) in bone homeostasis has been gradually revealed^6^, ^7^: Histone modifications such as H3K27 demethylase KDM6B^8^ activate osteogenic genes *BMP2/HOXC6*, and H3K9me3 inhibits *SOX9*, leading to cartilage degeneration^7^. DNA methylation promoted osteogenic differentiation through *RUNX2/OSX* promoter demethylation^9^, while *SOST* hypermethylation or *receptor activator of NF-κB ligand (RANKL)* hypomethylation regulated bone formation and resorption, respectively^10^. Non-coding RNAs such as miR-204/211^11^ inhibit RUNX2, lncRNA HOTAIR/DANCR inhibits osteogenic genes through the PRC2 complex^12^, and MIAT promotes osteogenic differentiation through the miR-150/AKT axis^13^. Despite the spatiotemporal specific or bidirectional regulation (e.g., contradictory effects of H3K9me3^14^ at different stages of osteogenic differentiation), targeting epigenetic mechanisms (e.g., HDAC inhibitors, miR-214 antagonists) has demonstrated therapeutic potential in preclinical models, suggesting their translational potential in orthopedic diseases. Among them, histone acetylation and deacetylation dynamically change the chromatin conformation and regulate the spatiotemporal specificity of gene transcription, which has become a key switch in the regulation of bone homeostasis^15^.

**Figure 1:**
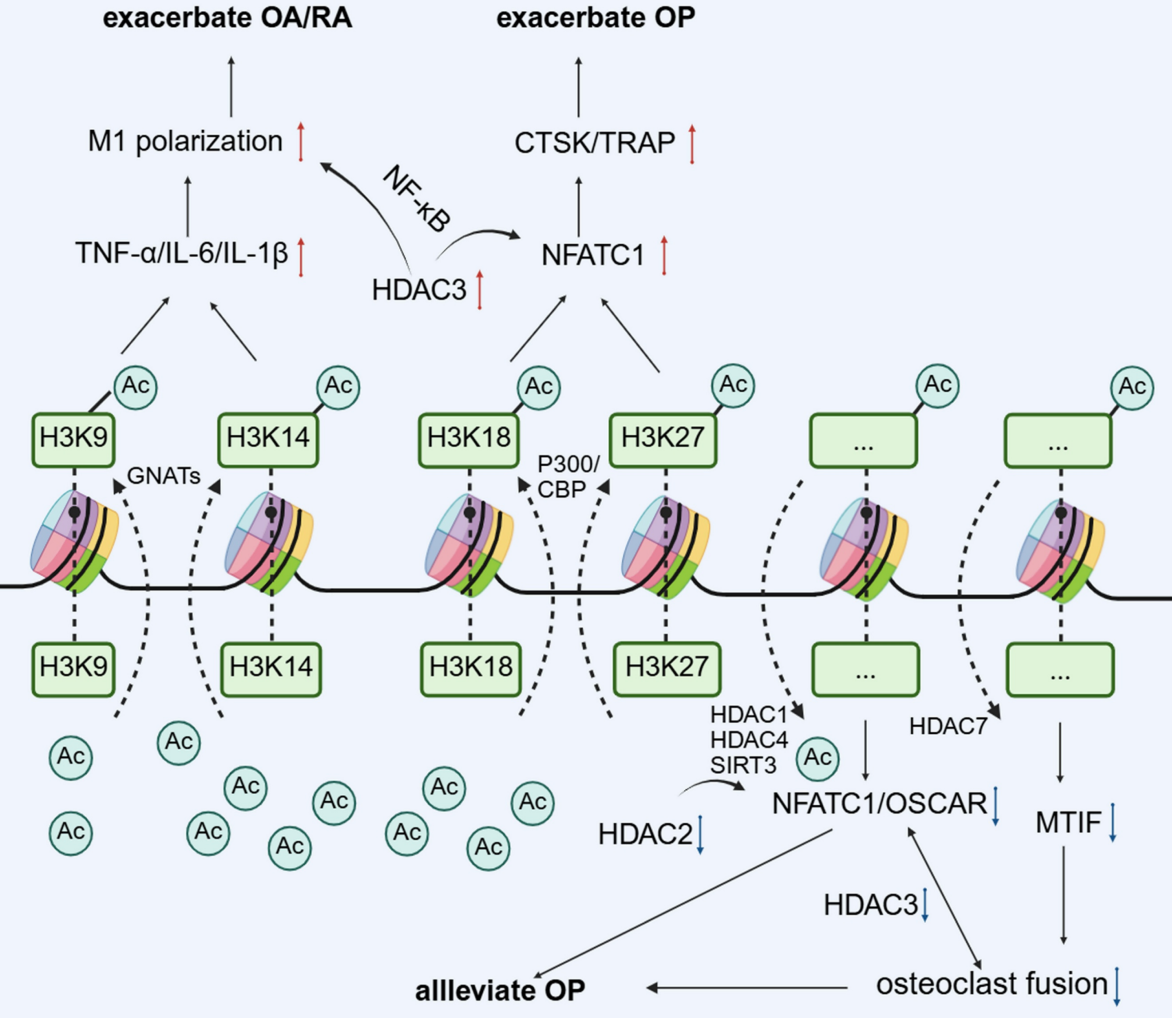
The changes of partial histone acetylation sites regulate gene expression and affect the biological effects of monocyte-macrophage.

The dynamic balance between histone acetylation and deacetylation is the core mechanism of epigenetic regulation [FIGURE 1]. Acetyl-CoA is the main acetyl donor, which regulates the chromatin structure and metabolic enzyme activity by transferring acetyl groups to lysine residues of histone or non-histone proteins through histone acetyltransferases (HATs/KATs). Its production pathways include glycolysis, fatty acid β-oxidation, and amino acid catabolism^16^, whereas nuclear ATP-citrate lyase-mediated acetyl-CoA synthesis^17^ is essential for histone acetylation in pluripotent stem cells. The acetylation level is regulated by metabolic status: the abundance of nutrient substrates (e.g., glucose, glutamine) affects acetyl-CoA production^18^, and AMPK signaling^19^ regulates the deacetylation activity through the phosphorylation of HDACs. HATs and HDACs can be assigned to different families in accordance with their structural characteristics, catalytic mechanism, and subcellular localization, as well as play specific regulatory roles in OC differentiation. Notably, the dysregulation of histone acetylation modification is disease-specific. For example, the *HDAC6* expression is significantly upregulated in monocytes from OP patients^20^, whereas in OA subchondral bone, OCs, and resident macrophages may activate the acetylation of the NF-κB promoter to release proinflammatory factors such as IL-6 and TNF-α via HATs^21^ aggravated the joint inflammatory microenvironment. These findings suggest that targeting HATs/HDACs may enable precise intervention of the monocyte-macrophage system by restoring the epigenetic homeostasis. Presently, a variety of HDAC inhibitors, such as SAHA and Tubastatin A, have exhibited osteoprotective effects in preclinical models^22^, ^23^, albeit their tissue selectivity and safety warrant further optimization.

### 1.1 Classification and function of HATs

HATs (“Writers”) facilitate the binding of transcription factors to DNA by catalyzing acetylation of the lysine residues in histone tails and neutralizing positive charges to relax the chromatin structure^24^. Based on the domain characteristics, HATs can be mainly divided into the following families:

#### 1.1.1 GNAT family (GCN5-related N-acetyltransferases)

The GNAT family members (e.g., GCN5, PCAF) preferentially acetylate histone H3K9 and H3K14 sites through conserved acetyl-CoA binding and substrate recognition domains^25^. Past studies have reported that GCN5 (KAT2A) promotes the differentiation of macrophages into a proinflammatory phenotype (M1) through epigenetic reprogramming, secreting inflammatory factors such as TNF-α and IL-6, which aggravates the pathological process of RA and OA. The inhibition of the GCN5 activity can reduce inflammatory bone destruction^26^. Reduced PCAF is manifested as impaired OC differentiation and decreased bone resorption activity, and this mechanism is related to the transcriptional inhibition of OC promoting transcription factors such as NFATC1^27^.

#### 1.1.2 MYST family (MOZ, YBF2/SAS3, SAS2, TIP60)

MYST family members (e.g., Tip60, MOF) mediate the acetylation activity through the MYST domain^28^. In inflammation and immune regulation, TIP60 can enhance the expression of TNF-α, IL-6, and other proinflammatory factors through acetylation of the P65 subunit of NF-κB, promote the polarization of M1 macrophages, and aggravate the inflammatory response of OA and RA. Scaffold proteins containing bromodomain and PHD finger, such as BRPF, can recruit MYST family to specific chromatin regions by recognizing acetylated histone marks, thereby regulating the transcriptional activity of related genes during OC differentiation. The selective inhibition of bromodomain protein can significantly block RANKL-induced OC differentiation, thereby indirectly suggesting that the MYST family may affect this process through acetylation modification^29^.

#### 1.1.3 P300/CREB-binding protein (CBP) family

The HAT/CBP and its closely related P300 protein, because of their considerable sequence homology and functional overlap and cooperation, are often referred to as a single entity (CBP/P300)^30^, which mainly acetylates H3K18 and H3K27 sites^31^. Past studies have demonstrated that P300/CBP may regulate OC differentiation and bone resorption activity through multiple mechanisms: 1) as a coactivator, its acetyltransferase activity enhances the transcriptional activity of NFATC1-AP-1 complex and promotes the expression of OC-specific genes such as *ACP5* and *CTSK*^32^; 2) upregulate the expression of P300/CBP in response to inflammatory signals such as IL-17A through the STAT3/miR-7-5p axis and synergistically amplify the microenvironment promoting OC differentiation^33^, ^34^; 3) upregulation of P300 may aggravate RANKL-NF-κB signaling and metabolic reprogramming such as HIF-1α-mediated glycolysis^35^, thereby accelerating the bone loss. Selective inhibitor CBP30 can block the function of the bromodomain of P300/CBP and inhibit OC activity, suggesting that targeting P300/CBP may be a potential strategy to intervene in bone resorption^33^.

### 1.2 Classification and function of HDACs

HDACs (“Erasers”), which restore the chromatin density by removing the histone acetyl groups, are classified into four classes based on cofactor dependence and sequence homology:

#### 1.2.1 Classical HDACs (class I, II, IV)

Class I HDACs (HDAC1/2/3/8) are enriched in the nucleus and participate in global chromatin modification^36^. For example, the primary role of HDAC1 in OCs is to act as a co-repressor. HDAC1 was recruited to the promoter regions of genes such as *NFATC1* and *OSCAR* to repress their expression^37^. The HDAC2 expression is increased during OC differentiation. The *HDAC2* expression is increased during the differentiation of bone marrow-derived precursor OCs induced by nuclear factor-κB ligand (RANKL) receptor activator^38^. Past studies have demonstrated that the knockdown of *HDAC2* in OCs not only inhibits OC differentiation but also prevents actin ring formation, fusion, and OC activity. In addition, HDAC2 promotes OC differentiation by activating AKT, and AKT phosphorylation and FOXO1 inactivation act as negative regulators of OC differentiation^38^. The role of HDAC3 is complex and contradictory. The inhibition of *HDAC3* can inhibit OC differentiation and OC marker genes *NFATC1*,* CTSK*, and *DC-STAMP*^39^. Meanwhile, HDAC3 promotes M1 polarization by inhibiting anti-inflammatory gene expression and activating NF-κB, whereas the knockdown of *HDAC3* enhances the M2 marker expression and bone healing^40^, ^41^, and the loss of 50% lipopolysaccharide (LPS)-induced inflammatory gene expression due to the deficiency of IFN-β/Stat1 pathway^42^. Class II HDACs (HDAC4/5/6/7/9/10) can shuttle between the nucleus and cytoplasm^43^. The knockdown of *HDAC4* in OCs led to enhanced OC gene differentiation and upregulation^23^. HDAC3 and HDAC7 have opposite effects on OC differentiation. The inhibition of *HDAC3*, similar to HDAC inhibitors, inhibits OC differentiation^44^, whereas the inhibition of HDAC7 accelerates OC differentiation^39^ and the overexpression of *HDAC7* in mouse bone marrow-derived macrophages (BMMs) inhibited OC precursor (OCP) fusion by inhibiting the *MITF* transcriptional activity^45^. HDAC7 plays a different role in macrophage inflammation, as reflected in the amplified TLR signaling through the HIF-1α pathway and the promotion of the inflammatory response^46^. HDAC9 reduces osteoclastogenesis by downregulating the *RANKL* expression while enhancing the release of anti-inflammatory factors such as IL-10 through the inhibition of M1 polarization and the promotion of M2 phenotype^47^. Class IV HDAC11 inhibits OC differentiation and exerts anti-inflammatory effects indirectly by regulating the expression of* IL10*^48^, ^49^.

#### 1.2.2 Class III HDACs (Sirtuin family)

Sirtuins (SIRT1-7) depend on NAD⁺ to participate in energy metabolism and stress response. SIRT1 inhibits OC differentiation by deacetylating NF-κB *P65* and FOXO family proteins such as FOXO1/3/4^50^ and inhibiting the release of TNF-α and IL-6, reducing the inhibition of inflammation on osteoblasts (OBs)^51^, ^52^, and activating PPARγ, inducing M2 phenotype, and secreting transforming growth factor β (TGF-β), IGF-1, and other pro-repair factors. Its agonist, resveratrol, dose-dependently alleviated bone loss in a postmenopausal OP model^53^. The loss of SIRT3 increased the expression of OC gene markers OSCAR, NFATC1, and ATP6V0D2^54^, suggesting that SIRT3 is a negative regulator of OC differentiation. Metabolic factors and REDOX signaling intersect with epigenetic regulation in OCs. For example, reagents that modulate cellular NAD+ levels can affect the activity of sirtuins, which are not only essential for deacetylation but also act as metabolic sensors. The activation of deacetylases such as SIRT1 is associated with enhanced OB differentiation and reduced OC production, linking the metabolic status to epigenetic regulation^55^, ^56^. Future therapeutic strategies may incorporate metabolic interventions, such as NAD+ precursor supplementation, in combination with HDAC inhibitors or HAT activators, so as to achieve a more comprehensive restoration of bone homeostasis.

### 1.3 Classification and function of the bromodomain

The bromodomain proteins act as epigenetic “Readers” that recognize acetylated lysines in histones and transcribed proteins. Bromodomain is the only specialized domain that specifically binds to acetylation marks^57^ and plays an important role in chromatin-based cellular processes, including gene transcription and chromatin remodeling. BET family members (BRD2/3/4/BRDT), such as BRD4, promote the phosphorylation of RNA polymerase II by recruiting CDK9 kinase, which drives the expression of proliferation/inflammation-related genes such as c-MYC^58^. The knockdown of BET inhibited the expression of proinflammatory cytokine-induced catabolic factors^59^. BRD4 promoted RANKL-induced OC differentiation and bone resorption by binding to acetylated histones in the promoter regions of *NF-κB*, *NFATC1*, and *FOS* and enhancing their transcriptional activity^60^. In contrast, BRD9 negatively regulates OC differentiation by activating the FOXP1-STAT1-IFNβ-signaling axis, and its agonist alleviates steroid-induced osteonecrosis of the jaw and acute bone loss^61^. At the level of inflammatory macrophages, bromodomain-containing proteins regulate the balance of M1/M2 polarization of macrophages by recognizing histone acetylation. BRD4 binds to acetylated histones in the promoter regions of proinflammatory genes (TNF-α and IL-6), amplifies NF-κB signaling, and promotes M1 polarization (i.e., a proinflammatory phenotype). The BET inhibitor could block this pathway and inhibit the inflammatory response^62^. Bromodomain proteins affect the JAK/STAT, HIF-1, and other pathways through epigenetic reprogramming, ultimately determining the metabolic phenotype and function of macrophages^63^.

### 1.4 Synergistic and antagonistic regulatory networks of HATs/HDACs

HATs and HDACs interact through complex epigenetic regulatory mechanisms and jointly regulate gene expression. The competitive binding of P300/CBP to HDAC3 can affect the activity of transcription factors such as NFATC1, thereby regulating the expression of inflammation-related genes. This mechanism plays an important role in OC differentiation and the pathogenesis of OA^64^. Under the pathological conditions of inflammatory bone disease, functional imbalance of HATs and HDACs can lead to abnormal release of inflammatory factors. HATs promote the expression of proinflammatory factors such as IL-6 and TNF-α by acetylating the κB promoter, exacerbating the joint inflammatory microenvironment^65^. In addition, the abnormal activation of HDACs inhibits the expression of anti-inflammatory genes through deacetylation, which further aggravates the inflammatory response. HDAC inhibitors such as TSA and SAHA can reduce the expression of inflammatory factors and modulate inflammatory responses by inhibiting the activity of *NF-κB* transcription factors in mouse models^66^.

### 1.5 Function of HATs and HDACs in non-histone protein modifications

Although histone acetylation governs the “readability” of the genome, the acetylation of non-histone proteins governs the direct activity, stability, and interaction of the proteome itself. With thousands of non-histone proteins now identified as targets for acetylation, this post-translational modification is recognized as a major regulatory mechanism comparable in scope to phosphorylation. Acetylation of the same protein may have different effects, such as activation, inhibition, or no effect, depending on the site, which warrants experimental verification^67^. A prime example of non-histone acetylation's role is the modification of NFATC1. This acetylation event is critical for NFATC1's transcriptional activity^68^, and HDAC5 deacetylates and inhibits it. Although the functions of non-histone modifications are important, the mechanism, specific targets, and functional consequences of non-histone modifications in these processes remain unclear, and the related studies are scarce. Further research is thus needed to clarify the common and individual roles of non-histone acetylation modifications in gene transcription regulation and protein function changes in order to form a systematic theory.

## 2. The regulatory network of histone acetylation homeostasis and OC (OC) differentiation

### 2.1 The staged dynamic process of OC formation

OCs originate from hematopoietic stem cells (HSCs) in the bone marrow, and their differentiation process is strictly spatiotemporally regulated. In the bone marrow microenvironment, HSCs are driven by granulocyte-macrophage colony-stimulating factor (GM-CSF) and macrophage colony-stimulating factor (M-CSF) to differentiate into the monocyte-macrophage cell line^69^, ^70^. Monocytes bind to RANKL, secreted by OBs or stromal cells via the RANK receptor, to trigger downstream signaling. They then differentiate into OCPs, which fuse to form multinucleated OCs and release osteolytic enzymes, such as tartrate-resistant acid phosphatase (TRAP) and cathepsin K (CTSK). These precursors ultimately mature into functional OCs.^71^.

### 2.2 Core signaling pathways of OC differentiation

The RANKL/RANK/NF-κB pathway is the main driver of OC differentiation. RANKL activates RANK and recruits the IκBα kinase (IKK) complex via TRAF6 (TNF receptor-associated factor 6), which causes phosphorylation and degradation of IκB protein, releasing NF-κB into the nucleus to initiate transcription of target genes^72^. Of these, NFATC1, a pertinent transcription factor, forms a positive feedback loop via the synergistic action of NF-κB and AP-1 (C-FOS/C-JUN). This loop drives the expression of OC-specific genes, such as matrix metalloproteinase 9 (*MMP9*), cathepsin K (*CTSK*), and acid phosphatase 5(*ACP5*)^73^. In addition, the MAPK (ERK/P38) and PI3K-AKT pathways amplify differentiation signals by regulating NFATC1 stability and cell survival signals^74^. RANKL can induce NFATC1 acetylation and enhanced stability via HATs. HATs, such as P300 and PCAF, can acetylate NFATC1 under RANKL stimulation, thereby stabilizing the NFATC1 protein, enhancing its transcriptional activity, and promoting OC differentiation. HDAC inhibitors increase RANKL-mediated acetylation of NFATC1, whereas *HDAC5* overexpression reduces its acetylation, stability, and transcriptional activation activity. These findings demonstrate that the balance of HAT and HDAC activity is crucial for regulating NFATC1 levels^75^.

### 2.3 The dynamic regulation of histone acetylation/deacetylation during differentiation

**Figure 2:**
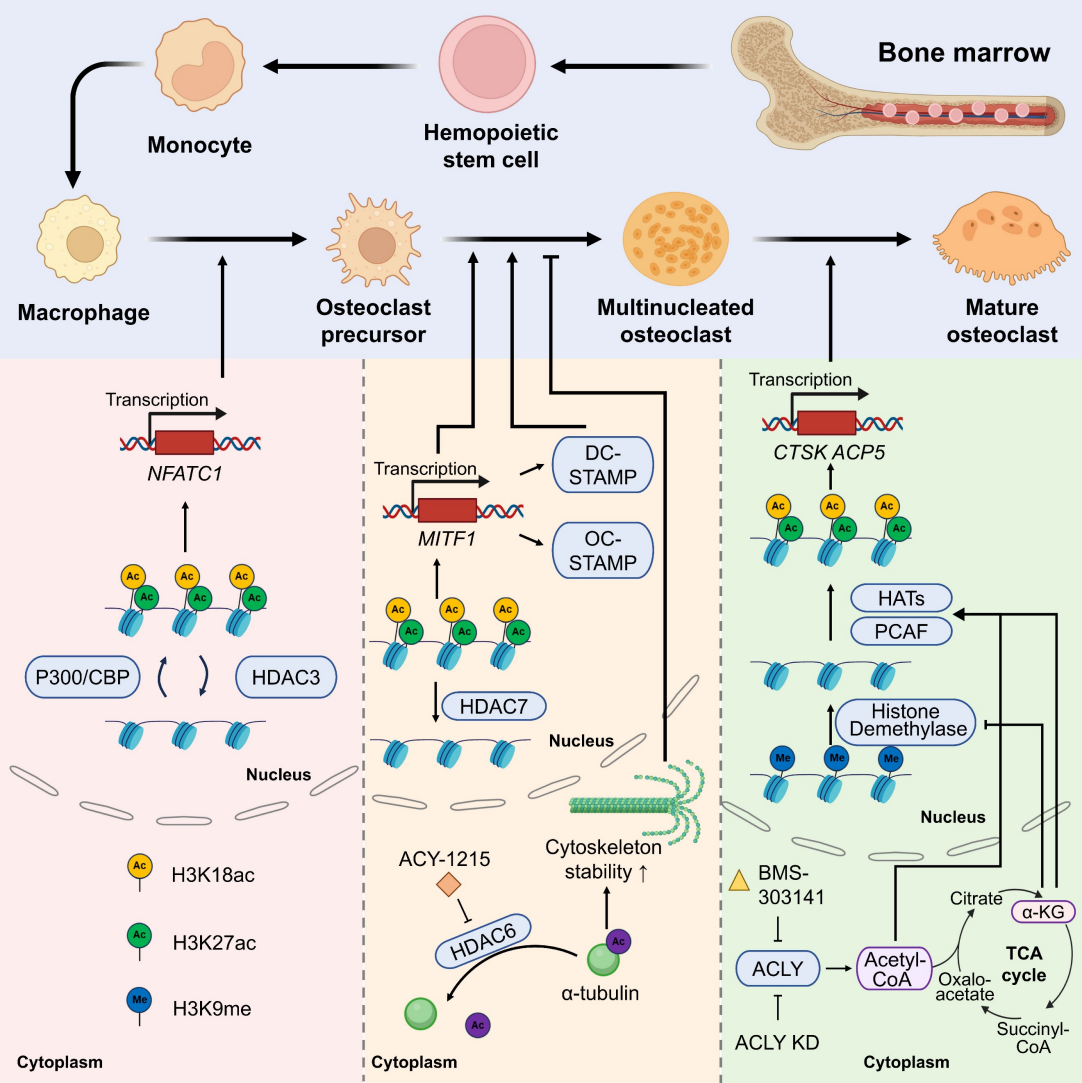
The scheme of roles of histone acetylases/deacetylases in different stages of osteoclast differentiation.

The proper execution of osteogenesis requires the dynamic regulation of chromatin structure to precisely control gene expression programs at various stages of differentiation. This dynamic regulation is predominantly achieved via the reversible action of HAT and HDAC, and this epigenetic modification is temporally and spatially regulated during OC differentiation to ensure adequate cellular function and homeostasis [FIGURE 2].

#### 2.3.1 Early stage: activation of NFATC1 by HATs/HDACs

During the transition of monocytes to OCPs, HATs stimulate the transcription of OC promoting genes by opening the chromatin structure. For instance, P300/CBP regulates OC differentiation and bone resorption activity via the acetylation of histone H3K18 and H3K27^27^, ^76^. Acetylation events in the promoter regions of OC markers enhance the transcription of genes such as *NFATC1* (a master regulator driving OC generation) and other OC-specific genes^14^, ^77^. By contrast, class II HDACs (such as HDAC4) inhibit NF-κB activity via deacetylation, and their knockdown results in the excessive differentiation of OCs^39^.

#### 2.3.2 Mid stage: acetylation modification regulates cell fusion and migration

During the nucleation stage of OCPs, no studies have reported that HATs/HDACs are directly related to the fusion-related genes *DC-STAMP* and *OC-STAMP*, but NFATC1 acts as an upstream factor to promote fusion-related genes. Furthermore, the overexpression of *HDAC7* in mouse BMMs inhibited OCP fusion by inhibiting *MITF* transcriptional activity^45^. HATs/HDACs may exert an indirect effect on *DC-STAMP* and *OC-STAMP*, which warrants further investigation. HDAC6 regulates cytoskeleton dynamics by deacetylating α-tubulin and inhibiting its activity (e.g., using the inhibitor ACY-1215), substantially reducing the migration and fusion efficiency of OCs^78^.

#### 2.3.3 Late stage: metabolic-epigenetic coupling regulates osteolytic function

Metabolic reprogramming is intricately associated with histone acetylation. α-Ketoglutaric acid (α-KG), a tricarboxylic acid (TCA) cycle intermediate, not only acts as a substrate of HATs to promote histone acetylation but also indirectly inhibits OC differentiation by inhibiting H3K9 demethylase activity^79^, ^80^. Moreover, the SIRT family of NAD^+^-dependent deacetylases, such as SIRT1 and SIRT6, affects OC activity by regulating related pathways, such as NF-κB and RANKL signaling, of which SIRT6 deficiency considerably augments OC generation and leads to OP^81^. ACLY was gradually activated during RANKL-induced BMM differentiation into OCs. Knockdown of *ACLY* or treatment with BMS-303141 significantly reduced nuclear and cytoplasmic acetyl-CoA levels in BMMs and OCs and inhibited OC formation. In addition, BMS-303141 inhibited OC formation and prevented ovariectomized (OVX) -induced bone loss *in vivo*^79^.

## 3. Homeostasis of histone acetylation regulates the polarization of macrophages

### 3.1 Proinflammatory and anti-inflammatory

Macrophages differentiate into two functionally distinct subtypes in response to microenvironmental stimuli. The M1 type is activated by interferon (IFN)-γ/LPS via the TLR4/NF-κB pathway^82^, secretes proinflammatory cytokines such as IL-6 and TNF-α, and promotes RANKL-mediated bone resorption in OP^83^. Enhanced MMPs mediated cartilage degradation in OA^84^. The M2 type is induced by IL-4/IL-13 via the STAT6/PPAR-γ pathway, which secretes anti-inflammatory factors such as IL-10 and TGF-β to promote osteogenic differentiation and cartilage repair facilitated by bone morphogenetic proteins (BMPs)^85^. Under inflammatory conditions, stimuli such as LPS or IFN-γ trigger signaling cascades that lead to the increased recruitment of HATs, including P300, to the promoters of inflammatory genes. The resulting hyperacetylation of histone tails, particularly at residues such as H3K27 and H4K16, facilitates the binding of transcription factors, such as NF-κB and STAT, thereby driving the expression of proinflammatory mediators. By contrast, HDAC counteracts this process by removing the acetyl groups, leading to chromatin condensation and reduced expression of these inflammatory genes. Histone modifications (e.g., H3K4 acetylation) promote M1 polarization by activating transcription factors (e.g., STAT1 and NF-κB). Several HDAC isoforms, such as HDAC3 and HDAC6, as well as class IIa family members, have been implicated in maintaining the proinflammatory cellular environment by inhibiting genes related to anti-inflammatory responses. Conversely, the inhibition of class IIa HDACs by agents such as TMP269 has been observed to drive M1 macrophage polarization, thereby intensifying the inflammatory response in injury models^86^. HDAC3 establishes a comprehensive inflammatory gene expression program in macrophages, and its loss or inhibition is associated with enhanced bone healing, resulting in a shift toward an M2-like repair phenotype^47^. These effects underscore the crucial role of histone acetylation dynamics in regulating the cellular functions of macrophages during inflammation and repair.

### 3.2 Crosstalk among metabolism, polarization, and histone acetylation

**Figure 3:**
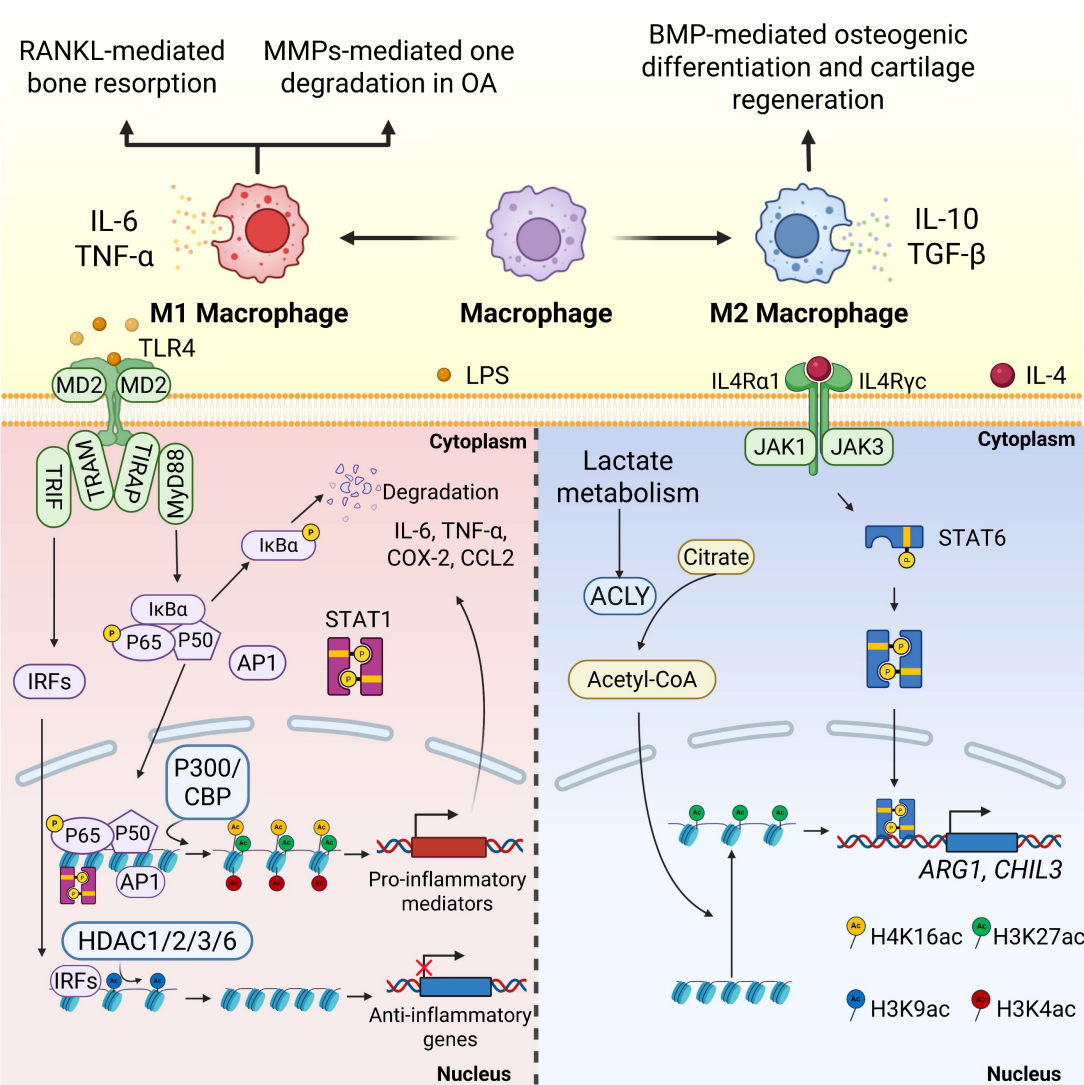
Histone acetylation is involved in common pathways for macrophage polarization in orthopedic disorders.

Recent studies have revealed the intricate interaction between cellular metabolism and histone acetylation, especially in macrophages. Metabolic substrates, such as acetyl-CoA, function as acetyl donors for HAT-mediated reactions, directly connecting the cell's energy status to its epigenetic landscape^87^. In proinflammatory M1 macrophages, increased glycolysis enhances acetyl-CoA levels, resulting in the histone acetylation of genes that drive inflammation. Conversely, M2 macrophages, which mainly depend on oxidative phosphorylation, exhibit different histone acetylation patterns that promote anti-inflammatory gene expression^87^. This metabolic-epigenetic link may be particularly significant in orthopedic conditions, where systemic metabolic issues, such as metabolic syndrome and diabetes, exacerbate inflammation and hinder bone healing. In regulating macrophage polarization, lactate metabolism boosts acetyl-CoA production via ACLY activation, leading to H3K27 acetylation and indirectly enhancing the expression of M2-associated genes (*Arg1* and *Ym1*) [FIGURE 3].

### 3.3 The monocyte-macrophage system interacts with osteoblasts to indirectly regulate bone injury repair

As the primary regulator of bone injury repair, macrophages are involved in the entire process of inflammation initiation, tissue remodeling, and bone regeneration. Regarding dynamic phenotypic switching, in the early stage of injury, infiltrating M1 macrophages clear necrotic tissue and stimulate angiogenesis by secreting TNF-α, IL-1β, and other proinflammatory factors. By contrast, during the middle and late stages of repair, they switch to the M2 phenotype, secreting IL-10, TGF-β, and other factors to suppress inflammation and promote osteogenic differentiation and matrix mineralization^88^, ^89^. CD169⁺ OsteoMacs cover >75% of the OB surface and support the mineralization of OBs via direct contact or the separation of BMP-2, platelet-derived growth factor (PDGF), and other factors. Depletion of OsteoMacs results in the loss of osteogenic function^90^, ^91^. Simultaneously, macrophages recruit mesenchymal stem cells via chemokines such as CCL2 and CCL5^92^ and activate the WNT signaling pathway to promote their differentiation into OBs^93^. A timely M1 to M2 conversion is crucial for bone regeneration, and an imbalance can lead to chronic inflammation or delayed repair^94^, ^95^. Persistent inflammation and impaired bone healing occur in aged individuals owing to disrupted M1 to M2 switching^96^, ^97^.

Histone acetylation is involved in the process of bone repair via the following mechanisms: First, HATs (such as P300/CBP) are recruited to the promoter regions of the key osteogenic transcription factors RUNX2 and OSTERIX, and their transcriptional activity is enhanced via H3K27ac modification^98^ to promote bone matrix synthesis. Second, the increased level of histone acetylation in macrophages considerably augments the secretion of osteogenic factors such as BMP-2 and PDGF, directly activating the differentiation pathway of OBs^91^, ^93^.

### 3.4 The double-sided effects of HAT/HDAC and NF-κB pathways in OC differentiation and macrophage polarization

Unsurprisingly, the same core signaling molecules (e.g., NF-κB) and epigenetic modifications (e.g., histone acetylation) can mediate markedly distinct fates in macrophages. This mediation is governed not by a single factor, but by the interaction of different cell subtypes, the signaling microenvironment, and epigenetic programming. The key difference lies in the varying cell fates and functions. OC differentiation is a terminal process during which macrophages fuse to form multinucleated OCs that can resorb bone, driven by M-CSF and RANKL. Conversely, M1 polarization is a process of functional activation in which macrophages develop a proinflammatory and bactericidal phenotype after stimulation by IFN-γ and LPS, while maintaining their cellular identity.

Various upstream stimuli, such as RANKL and LPS, direct NF-κB to bind to different genomic enhancer regions and recruit specific cofactors. This binding results in distinct acetylation patterns that ultimately influence the activation of either NFATC1 or inflammatory factor genes. The NF-κB p65 subunit undergoes numerous posttranslational modifications, with acetylation at specific lysine residues serving as a functional code that modulates its activity^99^, ^100^. The different signaling pathways triggered by LPS and RANKL result in unique interactions between specific HATs and HDACs and the p65 subunit. This interaction creates diverse p65 acetylation patterns that influence its target gene selection. The M1-polarizing signal encourages hyperacetylation at K221/K310, enhancing inflammatory gene expression output^99^, ^101^, while a RANKL signal might induce a more nuanced and transient acetylation pattern sufficient for initial NFATC1 induction but not a full-blown inflammatory response.

HDACs play a central role in determining macrophage fate as their expression and activity significantly influence this process. These enzymes help regulate the balance between M1 and M2 phenotypes. HDAC3 specifically promotes M1 polarization, being vital for LPS-induced inflammatory gene expression and suppressing M2 polarization^102^, ^103^. Similarly, HDAC9 is expressed at high levels during M1 polarization, and its lack results in an amplified M2 phenotype^104^.

Macrophages exhibit remarkable heterogeneity and adaptability. Their functional characteristics are influenced by their origin (such as tissue-resident vs. monocyte-derived) and are critically dependent on signals from their microenvironment. This flexibility enables a single precursor cell, part of the monocyte-macrophage system, to assume a wide range of roles. Recent single-cell research has uncovered specific macrophage subpopulations, including “arthritis-associated osteoclastogenic macrophages,” which serve as different precursor pools for pathological OCs under inflammatory conditions^105^. This finding suggests that preexisting macrophage subpopulations, potentially characterized by distinct epigenetic landscapes, may be predisposed to a specific fate. Consequently, the core of their different differentiation paths is that the external signaling microenvironment directs the final functional outcome of NF-κB by activating internal epigenetic mechanisms within particular cell subsets.

## 4. Histone acetylation imbalance in the monocyte-macrophage system in bone diseases and targeted therapeutic strategies

**Figure 4:**
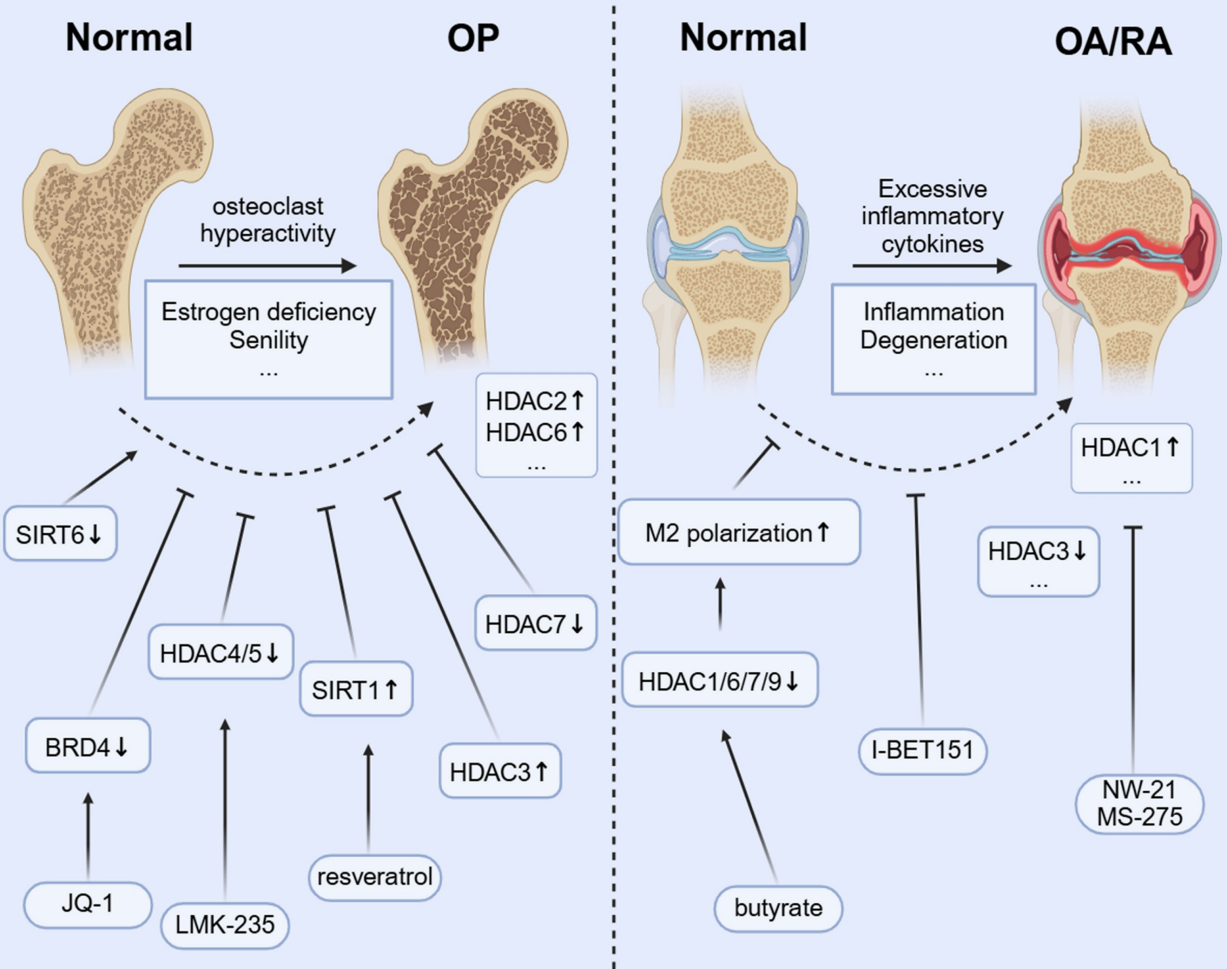
Histone acetylation modification affects bone metabolic balance and inflammatory response through epigenetic regulation, which is a new target for the treatment of osteoporosis and inflammatory bone diseases such as rheumatoid arthritis and osteoarthritis.

HDAC inhibitors have demonstrated the ability to diminish the dual therapeutic challenge of bone loss in preclinical models of RA and OP. This effect is achieved by regulating macrophage polarization and limiting OC-mediated bone resorption, making their use a promising approach for treating chronic inflammatory bone diseases^106^, ^107^ [FIGURE 4].

### 4.1 Osteoporosis

In OP, monocyte-derived OCs are the main regulators of bone homeostasis, and excessive activation of these cells leads to pathological bone resorption, which is a key feature of OP. As the only multinucleated giant cell responsible for bone resorption, their differentiation and activation are strictly controlled by the RANKL/RANK/OPG signaling axis, NF-κB, NFATC1, etc.^108^. The imbalance in the RANKL/OPG ratio owing to estrogen deficiency or aging leads to excessive OC differentiation. This results in systemic osteopenia, deterioration of bone microstructure, and a significantly higher risk of fractures^109^, ^110^. Conventional treatments for OC differentiation, such as bisphosphonates and denosumab, have demonstrated some effectiveness. However, their prolonged use can lead to side effects, including osteonecrosis of the jaw or other atypical fractures^111^, ^112^, and cannot reverse the damaged bone microstructure. Therefore, exploring novel regulatory mechanisms of OC differentiation is crucial for understanding bone metabolic diseases. Interventions targeting this pathway, such as HDAC inhibitors or PCAF activators, can suppress excessive OC activation by restoring acetylation balance, offering a promising new approach for treating OP.

Studies have found that *HDAC2* expression is increased in OP models, and its inhibition reduces OC differentiation and improves trabecular bone structure^113^. In addition, *HDAC7* deficiency results in enhanced OC activity and decreased bone mass by interfering with the MITF signaling pathway^45^. Nonetheless, the abnormal expression of class III HDAC *SIRT6* can affect OC function by regulating the metabolic-epigenetic network^114^, and its activator may have therapeutic potential.

The HDAC3-selective activator reduces OC differentiation by inhibiting the acetylation of NF-κB^44^. Resveratrol inhibits the RANKL-induced NF-κB signaling pathway and decreases IKK activity by activating SIRT1. This action prevents the acetylation and nuclear translocation of NF-κB-P65^115^. Broad-spectrum HDAC inhibitors (e.g., TSA and SAHA) increase histone acetylation globally^23^, ^116^, ^117^, potentially interfering with the epigenetic program required for OC differentiation, but their therapeutic window is narrow^118^, ^119^. Further development of subtype-selective inhibitors is necessary to strike a balance between efficacy and toxicity. For example, selective HDAC inhibitors (e.g., HDAC-1i and HDAC-2i) can dose-dependently reduce the number of OCs and the expression of key genes (e.g., *TRAF6*, *ACP5*, and *CTSK*) while inhibiting the release of proinflammatory cytokines. This finding suggests that targeting specific HDAC isoforms has therapeutic potential^120^ while avoiding side effects^121^. LMK-235, a selective HDAC4/5 inhibitor, not only inhibits OC differentiation but also promotes osteogenesis by regulating histone acetylation^122^. This dual effect highlights the importance of targeting the histone acetylation pathway in the monocyte-macrophage system to reduce excessive bone resorption and promote bone formation, especially in conditions such as OP and inflammatory bone loss RA^123^. The regulation of HATs has also received attention. The BET protein inhibitor I-BET151^124^ has been reported to block osteoclastogenesis by inhibiting the MYC-NFATC1 signaling axis and effectively attenuate inflammatory bone resorption and postmenopausal OP in a mouse model. JQ1 inhibits TLR/NF-κB signaling activation by preventing BRD4 accumulation at the NF-κB and NFATC1 promoters. This inhibition leads to a significant reduction in RANKL-induced expression of OC differentiation markers (TRAP and CTSK) and helps alleviate bone resorption^60^.

### 4.2 OA and RA

In monocytic macrophages, the dynamic regulation of histone acetylation is crucial for activating inflammatory genes and controlling macrophage polarization. This epigenetic process influences the switch between proinflammatory (M1) and anti-inflammatory (M2) phenotypes, playing a role in both the initiation and resolution of inflammation in orthopedic conditions^1^. In OA, while cartilage degeneration has conventionally been regarded as the primary pathology, recent research indicates that subchondral bone also plays a significant role in remodeling^125^, ^126^, with bone sclerosis and cystic lesions being key drivers of OA progression. Abnormal activation of OCs in the subchondral bone region not only accelerates local bone resorption but also exacerbates cartilage destruction and synovium inflammation by releasing proinflammatory factors such as IL-6 and TNF-α^127^. In RA, increased activity of synovial HDAC1 correlates with higher OC production, inflammation, and bone erosion. Inhibiting HDAC1 with new inhibitors, such as NW-21 and MS-275, has been shown to lower inflammatory cytokine levels, decrease key chemokines, including MCP-1 and MIP-1α, and reduce regulators of OC formation, such as TRAF-6 and NFATC1. These actions help reduce inflammation and prevent bone loss in arthritis models^106^. *HDAC3* expression and activity in peripheral blood mononuclear cells of patients with RA are significantly decreased, resulting in an increased level of histone H3 acetylation^128^. HDAC3 deficiency leads to increased acetylation of the NF-κB inhibitor protein (IκB), promotes the nuclear translocation of NF-κB, and upregulates inflammatory factors such as TNF-α and IL-6^129^. Synovial macrophages not only initiate the inflammatory response but also aid in resolving inflammation and facilitating tissue repair in OA^130^. When a joint is damaged, macrophages are attracted to the site of the injury. They influence chondrocytes and other cells by releasing inflammatory mediators and growth factors, which, in turn, impact joint repair and maintain tissue homeostasis^131^. Histone acetylation modulators can reprogram macrophage polarization, which is significant for inflammatory orthopedic conditions such as OA. Shifting macrophages from a proinflammatory M1 to a reparative M2 phenotype may help resolve chronic synovial inflammation, prevent cartilage breakdown, protect joint function, and slow disease progression^132^, ^133^. The microbial metabolite butyrate promotes IL-4-induced M2 polarization (an anti-inflammatory state) by inhibiting HDACs (HDAC1/6/7/9). This inhibition increases H3K9 acetylation and boosts STAT6 phosphorylation^134^, which has the potential to promote bone repair. I-BET151, a BET inhibitor, alleviates arthritic bone destruction and reduces arthritis severity by preventing the accumulation of inflammatory tissues without affecting the initial inflammatory phase^135^.

### 4.3 Bone repair

Macrophages are multifunctional immune cells that actively participate in bone repair by coordinating the initial inflammatory response and subsequent tissue remodeling^27^. In the initial inflammatory stage of bone healing, proinflammatory M1 macrophages clear debris and release cytokines that attract progenitor cells. When these macrophages switch appropriately to the anti-inflammatory M2 phenotype, they support tissue regeneration and remodeling, playing a crucial role in bone repair. Extended activation of M1 macrophages can lead to excessive inflammation and delay healing^132^. Increasing evidence highlights the crucial role of histone acetylation in regulating the macrophage phenotype and function during tissue repair processes^136^. By modulating chromatin accessibility and gene transcription, histone acetylation manages the fine balance between proinflammatory and anti-inflammatory macrophage repair states, which is vital for effective bone regeneration. Recent studies employing HDAC inhibitors suggest that inducing a hyperacetylated epigenetic state in macrophages improves inflammation resolution and supports tissue remodeling^137^. Moreover, metabolic and mechanical signals that influence acetyl-CoA production and activate HAT enzymes (such as P300/CBP) regulate macrophage responses. This forms an integrated signaling network that coordinates bone repair^138^. Future therapeutic approaches that precisely modulate histone acetylation in macrophages could accelerate fracture healing and improve outcomes for patients with complex bone injuries, all while minimizing unwanted side effects^41^. In summary, the emerging understanding of epigenetic regulation via histone acetylation in macrophages offers a promising new direction in bone regenerative medicine, emphasizing the need for further research with advanced molecular and translational studies^132^, ^139^.

### 4.4 Comprehensive treatment of histone acetylation imbalance in bone diseases

A therapeutic strategy that simultaneously targets macrophage polarization and OC differentiation has been demonstrated. The cornerstone of this dual-targeting approach is the shared origin of macrophages and OCs^140^. This common precursor, the monocyte, is a key upstream target for interventions aimed at regulating the effector functions of both cell types. By focusing on epigenetic programming early on, it may be possible to prevent the formation of harmful inflammatory macrophages and damaging OCs. To effectively utilize histone deacetylation targeting for synergistic benefits, interventions need to be carefully planned. This involves transitioning from general, broad-spectrum agents to more targeted and advanced techniques.

One approach involves designing a molecule that selectively inhibits a small, rational set of isoforms, such as a dual HDAC1/HDAC3 inhibitor. This type of compound could effectively reduce macrophage inflammation (via HDAC3) and osteoclastogenesis (via HDAC1) while avoiding the effects of other isoforms. Consequently, it may lessen the side effects commonly linked to pan-HDAC inhibitors^141^.

Another method is to develop targeted delivery systems. Bone-targeting delivery involves attaching the HDACi to a bone-homing compound. Bisphosphonates and tetracyclines are known for their strong affinity to hydroxyapatite, the mineral in bone. A bisphosphonate-HDACi conjugate would accumulate in regions with high bone turnover—exactly where inflammatory macrophages and OCs are most active—providing high local drug levels while reducing systemic exposure^142^. Macrophage-targeting delivery leverages the natural phagocytic activity of the monocyte-macrophage lineage. HDACi can be encapsulated within nanoparticles (e.g., liposomes and polymers) that are preferentially engulfed by these cells^143^. Further specificity can be achieved by decorating the nanoparticle surface with ligands that target receptors uniquely or highly expressed on monocytes and macrophages, such as the CD64 receptor^144^. Since OCs are derived from the same lineage, this approach can effectively deliver the therapeutic payload to OCPs as well. For example, Danshentsin A^145^ can improve bone repair by regulating HDAC3. The local administration of the BRD9 regulator, based on a silk protein hydrogel, to accurately alleviate osteonecrosis of the jaw^61^ provides a reference for this direction. Currently available investigational drugs are listed in TABLE 1.

## 5. Current challenges and prospects

Although strategies targeting HATs/HDACs have demonstrated promise in preclinical studies, their transition to clinical use encounters several obstacles. Primarily, the wide-ranging activity of HATs/HDACs could result in nonspecific chromatin remodeling. For example, HDAC inhibitors may activate both tumor suppressor and proinflammatory genes^146^, resulting in a trade-off between efficacy and toxicity. Second, the epigenetic regulatory networks of OCs, OBs, and chondrocytes interact in a complicated manner^147^, and tissue- or cell-specific delivery systems must be developed to avoid interfering with bone formation. In addition, the dynamic and reversible nature of histone modification requires drugs to exhibit continuous regulatory ability. Most of the existing small molecules have transient action^148^, ^149^, and long-acting agents should be developed to achieve stable intervention. Combining metabolomic analysis with epigenetic studies can help elucidate metabolic-epigenetic interactions that regulate macrophage function. Understanding how changes in acetyl-CoA levels and other metabolic intermediates affect histone acetylation in macrophages could reveal new therapeutic targets^87^.

In addition to histone acetylation of the monocyte-macrophage system, there are also nonhistone acetylation and other histone modification types: acetylation of NFATC1 improves protein stability^150^; Ezh2-mediated trimethylation of H3K27 (H3K27me3) promotes OC differentiation, and the demethylase Jmjd3 activates the gene table by removing H3K27me3 at the promoter regions of key genes such as *NFATC1*^151^. The newly discovered histone lactylation modification influences macrophage polarization. During initial immune activation (M1 phenotype), a glycolytic surge leads to lactate buildup; later, lactate promotes H3K18la modification, increasing anti-inflammatory gene expression (such as Arg1 and VEGF) and supporting the shift of macrophages toward the reparative M2 phenotype^152^. Research in these areas remains scattered and preliminary, with an incomplete evidence chain; therefore, additional experimental studies are necessary to develop a systematic understanding of the role of nonhistone acetylation and other histone modifications in regulating orthopedic disorders.

We propose several clinical prospects for regulating histone acetylation in monocyte-macrophage systems to treat diseases. (1) Developing precise targeted strategies: Using the CRISPR-dCas9 system to fuse HAT (e.g., p300) or deacetylase (e.g., HDAC3) domains for epigenetic editing at specific gene loci, and creating proteolysis-targeting chimeras along with more potent and isoform-specific HDAC and HAT inhibitors. (2) Investigating dynamic regulation pathways: Studying how cellular metabolic states (including acetyl-CoA levels) and signaling pathways (such as cytokine-mediated posttranslational modifications) influence acetylation to achieve spatiotemporal and adaptive regulation of the pathological environment. This strategy includes developing responsive drug delivery systems that release HAT/HDAC inhibitors only in response to specific pathological cues—for example, engineering nanoparticles to release their payload in low-pH environments typical of inflammation or active bone resorption by OCs. (3) Mapping protein-protein interaction networks (interactomes) of key HATs and HDACs in different cell types to identify unique cofactors and transcription factors that recruit them to their target genes, thereby conferring cell-type-specific functions. Collectively, these strategies aim to transition histone acetylation research into precise, dynamic, and cell-specific therapeutic approaches.

## Conclusion

Recent preclinical studies suggest that histone acetylation in the monocyte-macrophage system significantly influences inflammatory responses, polarized phenotypes, and osteogenesis processes, all of which are closely linked to the development of orthopedic diseases. Modulating histone acetylation via HDAC inhibitors and other epigenetic agents has been shown to decrease proinflammatory cytokine production, prevent pathological bone resorption, and encourage bone formation. Although dedicated clinical trials in this area are lacking, strong evidence from research on RA, OC differentiation, and metabolic-epigenetic interactions offers a compelling basis for further exploration of epigenetic targeting strategies for orthopedic disorders.

## Figures and Tables

**Table 1:** Small molecule compounds targeting histone acetylation modifications can be applied as potential therapeutic approaches for orthopedic disorders.

Disease type	Drug	Target cells	Molecular mechanism	Specific diseases	References cited
Bone resorption diseases	Quisinostat	Osteoclast	Inhibited the expression of *CFOS* and *NFATC1* and increased the acetylation of NF-κB-P65, thereby blocking its nuclear accumulation	Osteoporosis; Titanium particle-induced bone resorption	153, 154
	Puerarin	Osteoclast; osteoblast	Inhibited HDAC1 and HDAC3, regulated ALP, RUNX2, TRAP, etc. It also inhibited the inflammatory response and apoptosis, and reduced the expression levels of IL-6, TNF-α, COX2 and MMP-14 related to the inactivation of NF-κB signaling	Diabetic osteoporosis	155
	Resveratrol	Osteoclast; osteoblast	Activation of SIRT1 inhibited RANKL-induced NF-κB signaling pathway and reduced IκBα kinase (IKK) activity, thereby blocking the acetylation and nuclear translocation of NF-κB-p65	Senile osteoporosis	115, 156, 157
	Rosavin	osteoclast	Inhibition of HDAC1 promoted EEF2 expression, which in turn improved NF-κB and MAPK pathways	Osteoporosis	158
	Trichostatin A (TSA)	Osteoclast	HDACi, TSA significantly inhibited RANKL-mediated activation or induction of ERK, C-FOS and NFATC1. However, RANKL activation of JNK, p38, and NF-κB was not affected by TSA	Osteoporosis	159
	Entinostat (MS-275)	Osteoclast; osteoblast; M1 macrophage	Inhibition of NF-κB; Accelerated cell proliferation and ALP production to enhance osteoblast differentiation in MC3T3-E1 cells	Osteolysis of the skull	160-163
	N-methylpyrrolidone (NMP); N-vinyl-2-pyrrolidone (NVP)	Osteoclast; osteoblast; osteocyte	Low affinity BRD inhibitor, promoted osteogenesis, inhibited osteoclast, and inhibited sclerostin expression	Osteoporosis	164, 165
	(+)-ND	Osteoclast	BRD4 inhibitor, (+) -ND treatment inhibited MAPK and NF-κB signaling pathways	Osteoporosis	166
	JQ1	Osteoclast; osteoblast	A high-affinity BET protein inhibitor that inhibited the expression of the master osteoclast transcription factor NFATC1 and osteoblast transcription factor RUNX2.	Osteoporosis	167
	I-BET151	Osteoclast	The BRD inhibitor, I-BET151 acted by inhibiting MYC and targeting the newly described MYC-NFAT axis important for osteoclast generation	Osteoporosis	124
Inflammatory bone disease	SAHA	M1 macrophage	P21 expression induction, suppressed NF-κB nuclear accumulation	Rheumatoid arthritis	163
	Garcinol	M1 macrophage	Targeted PCAF inhibition with synergistic NF-κB and H3K9Ac blockade	Autoimmune arthritis	168

## Abbreviations

BMDMs: bone marrow-derived monocytes
ACLY: ATP citrate lyase
AS: ankylosing spondylitis
OP: osteoporosis
OA: osteoarthritis
RA: rheumatoid arthritis
HATs: histone acetylases
HDACs: histone deacetylases
GNAT: GCN5-related N-acetyltransferases
CBP: CREB-binding protein
OCs: osteoclasts
HSCs: hematopoietic stem cells
GM-CSF: granulocyte-macrophage colony-stimulating factor
M-CSF: macrophage colony-stimulating factor
OCPs: osteoclast precursors
TRAP: tartrate-resistant acid phosphatase
CTSK: cathepsin K
MMP9: metalloproteinase 9
TRAF6: TNF receptor-associated factor 6
α-KG: α-ketoglutaric acid
TCA: tricarboxylic acid
OVX: ovariectomized
